# Arthroscopic Synovectomy and Postoperative Assisted Radiotherapy for Treating Diffuse Pigmented Villonodular Synovitis of the Knee: An observational retrospective study

**DOI:** 10.12669/pjms.314.7383

**Published:** 2015

**Authors:** Wei Li, Xiaofei Sun, Jianning Lin, Wei Ji, Dike Ruan

**Affiliations:** 1 Wei Li, MD. Dept. of Orthopedics, Navy General Hospital, Beijing 100048, P.R. China; 2 Xiaofei Sun, MD. Dept. of Orthopedics, Navy General Hospital, Beijing 100048, P.R. China; 3 Jianning Lin, MD. Dept. of Orthopedics, Navy General Hospital, Beijing 100048, P.R. China; 4 Wei Ji, MD. Dept. of Orthopedics, Navy General Hospital, Beijing 100048, P.R. China; 5 Dike Ruan, MD, PhD. Dept. of Orthopedics, Navy General Hospital, Beijing 100048, P.R. China

**Keywords:** Knee joint, Pigmented villonodular synovitis, Arthroscopy, Radiotherapy, Recurrence

## Abstract

**Objective:**

This retrospective observational study aims to explore the treatment procedure and outcomes of arthroscopically assisted radiotherapy for diffuse pigmented villonodular synovitis (PVNS) of the knee joint.

**Methods:**

From September 2006 to August 2011, 28 diffuse PVNS patients were diagnosed and treated under arthroscopy. Twenty six underwent post-operative radiotherapy. All patients were followed up, and the average follow-up period was 54 months (range: 24 to 72 months).

**Results:**

All 26 patients who received external radiotherapy showed no recurrence at post-operative follow-up; The Lysholm knee joint function score increased from 54.3±9.0 at pre-operation to 71.2±6.7 at post-operation (paired t-test, t = −13.35, P< 0.01).

**Conclusions:**

Arthroscopic synovectomy is an ideal treatment for PVNS of the knee. Adjuvant post-operative external radiotherapy prevents the recurrence of diffuse PVNS.

## INTRODUCTION

Jaffe was the first to describe pigmented villonodular synovitis (PVNS) as a chronic proliferative disease that occurs in the joint, tendon and bursa synovium.^1^ According to Jaffe, PVNS is characterised by thickened and hyper-plastic synovia organised into villi and nodules, which lead to the deposition of intra-cellular haemosiderin pigments. This disease has two types: localised (giant cell tumour of the tendon sheath or nodular tenosynovitis) and diffuse (diffuse-type giant cell tumour or PVNS).^2^ Diffuse PVNS poses a significant risk of inadequate excision and recurrence, particularly in the knee joint.^3^ Diffuse PVNS also represents a medical challenge because of non-specific symptoms that delay the diagnosis with a high rate of recurrence.^4^ Multiple recurrences can also cause loss of joint and limb functions or amputation, malignant transformation and metastasis.

Magnetic resonance imaging (MRI) is usually used for PVNS diagnosis. However, histopathology is recognised as the gold standard for the final diagnosis of PVNS. Arthroscopic have been performed with variable success rates for PVNS treatment.^5^,^6^ Total arthroscopic synovectomy is technically demanding but can be advantageous. Adjuvant post-operative external beam radiation therapy for extensive diffuse and recurrent PVNS of the knee is also a reliable treatment method.^7^-^10^

However, the recurrence rate, chromosomal abnormalities and malignant transformation vary for diffuse PVNS patients subjected to different treatments. This retrospective study aims to evaluate the results of treatments with total arthroscopic synovectomy, followed by adjuvant radiotherapy.

## METHODS

The study was approved by the Ethics Committee of PLA Navy General Hospital (2012-A-066) and was conducted in accordance with the principles expressed in the Declaration of Helsinki. All patients were properly instructed and provided informed consent.

This retrospective study was conducted at the Navy General Hospital (Beijing, China). This study included 39 participants (25 males and 14 females) with knee PVNS, as confirmed by pathological examination of the shaved synovium from September 2006 to August 2011 (Table-I). The participants had a median age of 53 years (range: 39 to 66 years old). The disease course ranged from 4 months to 25 years, with an average of 5.5 years. Of the 39 cases, 20 involved the right knee and 19 involved the left knee. According to the eligibility criteria, all 39 participants were subjected to MRI to preliminary diagnose PVNS. Preliminary MRI diagnosis confirmed that 11 cases had the localised type and 28 had the diffuse type. In total, 30 patients presented joint swelling associated with floating patella syndrome and 9 patients presented no swelling; 28 patients had cavernous suprapatellar bursa. In addition, 32 patients experienced joint pain, and 21 patients had limited joint activities. All patients had normal body temperature. The pre-operative Lysholm knee score was 54.3±9.0 during the study period.^11^

**Table-I:** General data of patients.

Patient No.	Age (years)	Sex	Follow-up (months)	Variety	Lysholm Score (Pre)	Lysholm Score (Post)	Recurrence	Radiotherapy
1	51	F	36	Diffuse	45	70	No	Yes
2	43	M	60	Localised	69	75	No	No
3	60	M	66	Diffuse	42	70	No	Yes
4	46	M	72	Diffuse	55	73	No	Yes
5	49	F	54	Localised	68	75	No	No
6	62	M	36	Diffuse	60	77	No	Yes
7	55	F	54	Diffuse	42	61	No	Yes
8	52	M	60	Diffuse	47	71	No	Yes
9	50	M	66	Localised	60	81	No	No
10	57	F	60	Diffuse	55	79	No	Yes
11	49	M	66	Localised	70	77	No	No
12	39	M	30	Diffuse	60	71	No	Yes
13	41	F	54	Diffuse	43	70	No	Yes
14	58	M	72	Diffuse	42	60	No	Yes
15	53	M	60	Diffuse	54	77	No	Yes
16	55	M	66	Diffuse	55	81	No	Yes
17	66	M	48	Localised	60	70	No	No
18	46	F	60	Diffuse	42	66	No	Yes
19	48	M	54	Diffuse	47	61	No	Yes
20	50	F	54	Diffuse	51	79	No	Yes
21	51	M	60	Localised	56	60	No	No
22	50	F	36	Localised	57	70	No	No
23	62	M	30	Diffuse	51	66	No	Yes
24	53	M	48	Diffuse	65	74	No	Yes
25	57	M	24	Diffuse	55	77	No	Yes
26	45	M	48	Diffuse	60	73	No	Yes
27	63	F	66	Diffuse	51	57	No	Yes
28	58	F	66	Diffuse	55	72	No	Yes
29	50	M	60	Localised	70	78	No	No
30	47	F	54	Diffuse	57	73	No	Yes
31	53	M	54	Diffuse	42	71	Yes	No
32	52	M	60	Localised	70	75	No	No
33	50	F	42	Diffuse	55	74	No	Yes
34	61	M	60	Diffuse	43	60	No	Yes
35	59	F	48	Diffuse	60	79	No	Yes
36	48	F	66	Localised	66	70	No	No
37	55	M	54	Diffuse	44	57	No	Yes
38	62	M	60	Diffuse	51	71	Yes	No
39	50	M	54	Localised	43	77	No	No

### Arthroscopic treatment

**Fig.1:**
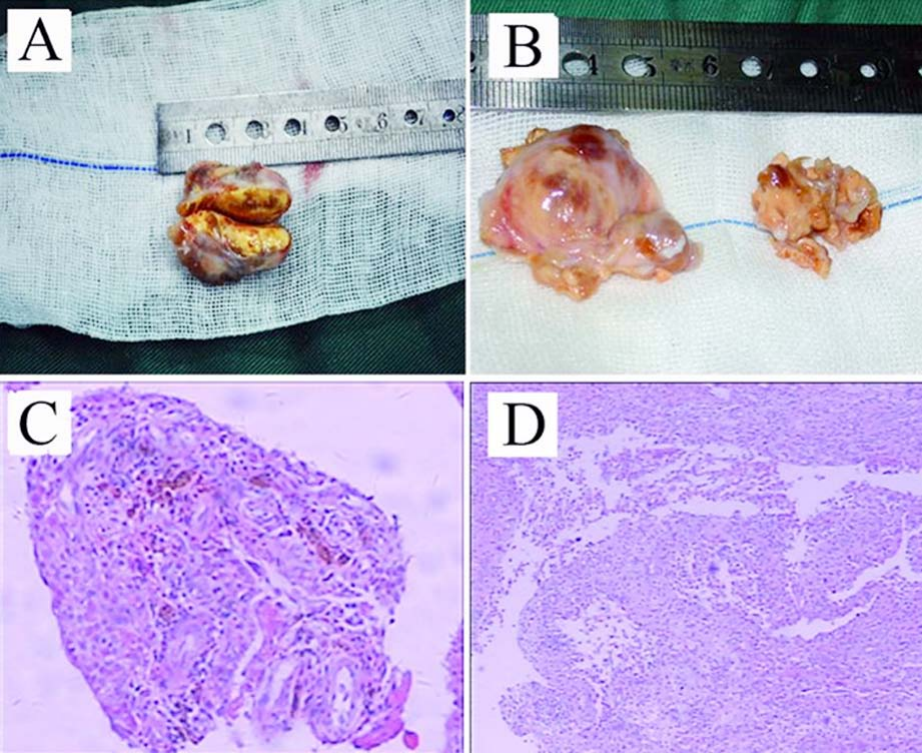
Morphosis and pathological section of PVNS: morphosis of (A) localised type and (B) diffuse type; pathological section of (C) localised type and (D) diffuse type.

**Fig.2:**
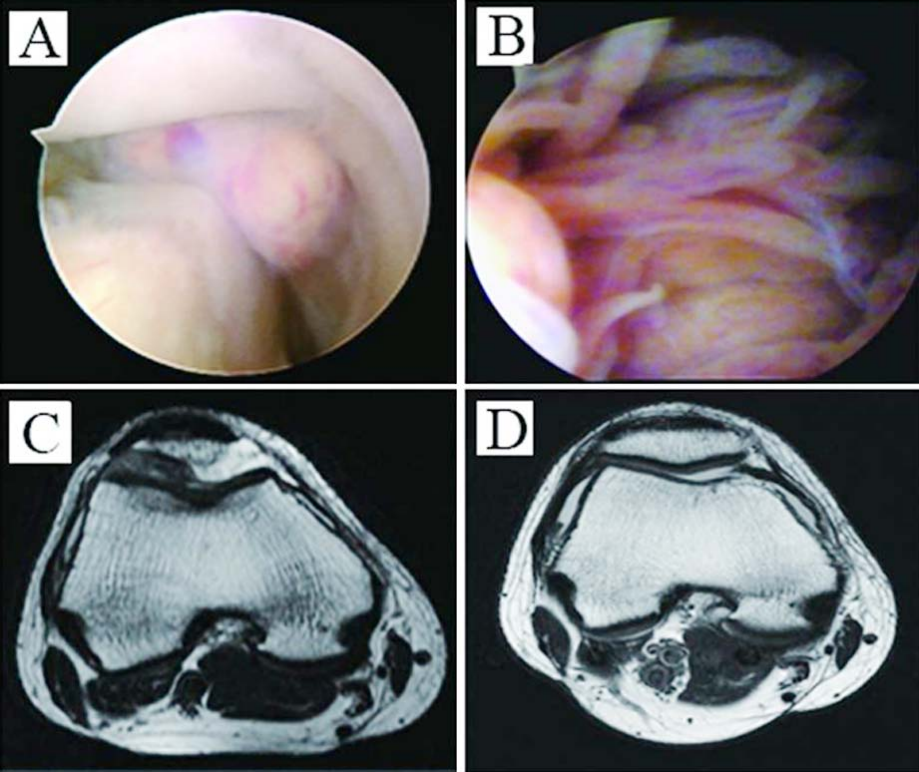
Intra-operative arthroscopic diagnosis for (A) localised type and (B) diffuse type; (C) pre-operative MRI examination; (D) MRI examination of the same patient at 5 years after the operation.

The patients were anaesthetised with Sevoflurane (induction: 5% in 100%O_2_; maintenance, 1.0% to 1.5%). The operation instruments included 30° and 70° arthroscopy, planing knife series, arthroscopic special electric knife and Arthrocare 2000 plasma operation system. Of the 39 patients, 36 were placed in full-course supine position (the affected knee can be hanging on the bed) and 3 were initially placed in supine position and then in prone position. The operation included anterolateral, anteromedial, medial and lateral approaches of bursae suprapatellaris. The localised (Fig.1A) and diffuse PVNS (Fig.1B) patients were gradually removed of lesion synovium with the planing knives as far as possible and were removed of the lesions that could not be reached by the planter. Then, cauterisation was carefully performed under the microscopic electric knife and Arthrocare 2000 plasma operation system. The operation sequence was inter-condylar fossa, anteromedial joint compartment, suprapatellar bursa and then external and internal crypt. The intra-operative arthroscopic diagnoses for localised and diffuse PVNS are shown in Figs. 2A and 2B, respectively. The Arthrocare 2000 plasma operation system was principally used to gradually isolate diffuse nodules from the synovium and remove them from the joint cavity block by block using a pith nucleus clamp. All patients underwent post-operative pathologic examination, and the diagnosis was confirmed after histopathological examination of the shaved synovium. The pathological diagnoses for the patients with localised and diffuse PVNS are shown in Figs. 1C and 1D, respectively.

### Post-operative radiotherapy

Without placement of post-operative drainage, the patients were allowed to perform quadriceps femora exercise at 24 hour after the operation and limb loading at 48 h to 72 hour after the operation. The sutures of arthroscopic incision were removed after seven days.

After wound healing, 26 of the diffuse PVNS patients received whole post-operative radiotherapy at a dose of 2000 cGy to 3000 cGy. The irradiation was performed 10 times, once every other day. However, 2 of the diffuse PVNS patients did not complete the radiotherapy; they only completed 600 cGy. All of the localised PVNS patients did not undergo external radiotherapy.

All 39 patients were followed up, and the average follow-up period was 54 months (range: 24 to 72 months). We recruited all participants who returned to the hospital for evaluation of Lysholm knee scores and MRI examination during follow-up to reduce bias. The baseline characteristics and recurrence rates of the patients were also collected. Researchers from our department collected all study data.

### Statistical analysis

Descriptive data were shown in this observational study. The pre-operative and post-operative Lysholm knee scores were quantitative variables that were analysed using Stata 13.0 statistical software. The variables were compared with *t*-test. Average values were presented as mean±SD, and *P*< 0.05 indicated statistical significance.

## RESULTS

### Baseline characteristics of patients

During the operation, joint effusion was extracted from 31 patients. The fluid volume ranged from 5 mL to 80 mL, with an average of 35 mL. Dark red and yellow turbid liquid were observed in 25 and 3 patients, respectively. In the 28 diffuse PVNS patients, villus overgrowth might fracture into the free villus body in the bursa suprapatellar. Three of these patients showed multiple brown diffuse nodules that were connected to occupy the synovial surface. Some nodules with a diameter of 1.5 cm to 5 cm protruded of the articular capsule and formed enclosed mass in the external suprapatellar bursa or popliteal soft tissues. All cases were confirmed by pathology. All 39 PVNS patientsreceived full-course knee arthroscopic synovium resection. The average operation time was 70 minutes (40 minutes to 150 minutes).

### Clinical Outcomes

All 26 diffuse PVNS cases who received external radiotherapy showed no recurrence. The pre-operative MRI examination results are shown in Fig.2C, and the MRI findings of the same patients at 5 years after the operation are shown in Fig.2D. Two patients who did not complete post-operative radiotherapy had in situ recurrence after 6 months, as verified by MRI. Thus, they underwent arthroscopic resection again plus post-operative radiotherapy; they showed no recurrence when post-operative follow-up (Table-I). The Lysholm knee score of all patients increased from 54.3±9.0 at pre-operation to 71.2±6.7 at post-operation (paired *t*-test, *t* = −13.35, *P*< 0.01). The limited joint function was improved significantly and the joint flexion was more than 120° after the operation. No complications, such as infection and vein thrombosis, were noted.

## DISCUSSION

Finis reported that PVNS is caused by continuous proliferation of local lesions when apoptosis-related molecules are inhibited.^12^ PVNS is a chronic proliferative tumour that occurs in the joint, tendon and bursa synovium, and can lead to bone invasion; however, the pathogenesis of this disease remains unclear. The biological behaviour and clinical manifestations of this disease are diverse and complex, with high recurrence rate. Multiple recurrences can cause the loss of joint function, malignant transformation and metastasis. The prognosis of malignant PVNS is poor.^13^ The WHO defined PVNS as a tumour lesion of soft tissue and bone tumour pathology and genetics. 14

The diagnosis of PVNS is difficult because of the lack of clinical characteristics. Joint puncture of brown or dark red joint fluid may help in the diagnosis of this disease. In the current study, dark red effusion was extracted in 25 of the 39 patients, and the remaining effusion was yellow or none. However, the confirmed diagnosis depends on pathology, and MRI examination is helpful in diagnosing the disease. The MRI results showed visible long T1 and T2 signals in different degrees of joint effusion and synovium diffuse hyperplasia.

The results of this study showed the following advantages of arthroscopy for PVNS treatment: easy biopsy for diagnosis and for determining the scope and type of treatment; simultaneous treatment of associated lesions; minimal invasion; quick recovery; and minimal complications.

The operation alone may not completely remove the synovial tissue; therefore, combination with other therapeutic measures was advocated to improve the effect of the operation. Blanco et al. performed arthroscopic anterior synovectomy and adjuvant external beam radiotherapy.^15^ They obtained a rate of 86% and a recurrence rate of 15.8% after ultrasonic inspection or clinical confirmation. Shabat et al. conducted post-operative intra-articulation radiotherapy after operation resection on 10 cases of PVNS, with a mean follow-up for 6 years.^16^ No recurrence was observed in all 10 cases. Chin et al. considered that the final effect of intra-articulation radiotherapy, although effective in removing small lesions, depends on the thorough resection of the lesion.^17^

In the current study, 26 of the diffuse PVNS patients underwent radiotherapy after surgery. These patients incurred no radioactive skin lesions during the follow-up and exhibited no local recurrence. The 11 localised PVNS patients were treated with simple arthroscopic synovection and without post-operative radiotherapy. These patients also showed no recurrence. Post-operative joint function was significantly improved compared with pre-operative joint function. This study also has several limitations. Firstly, this study was conducted at a single hospital. Secondly, this retrospective, observational study was limited by the nature of its design.^18^ Potential biases may arise from some factors related to both surgeons and patients. However, to reduce bias, we recruited all participants who returned to the hospital for evaluation of Lysholm knee scores and MRI examination during follow-up.

## CONCLUSIONS

The results of this study proved that arthroscopic synovectomy is ideal for treating PVNS of the knee joint. Appropriate amount of adjuvant post-operative external radiotherapy prevents the recurrence of diffuse PVNS after operation. We conclude that arthroscopic treatment of PVNS has the advantages of minimal complications, minimal invasion and fast post-operative recovery. The arthroscopic operation can be performed in diffuse PVNS patients, the appropriate dose of adjuvant post-operative external radiotherapy is also important to reduce the recurrence rate.
